# Osteopontin contributes to late-onset asthma phenotypes in adult asthma patients

**DOI:** 10.1038/s12276-020-0376-2

**Published:** 2020-02-03

**Authors:** Hoang Kim Tu Trinh, Thuy Van Thao Nguyen, Seo-Hee Kim, Thi Bich Tra Cao, Quoc Quang Luu, Seung-Hyun Kim, Hae-Sim Park

**Affiliations:** 1 Department of Allergy and Clinical Immunology, Ajou University Medical Center, Suwon, South Korea; 2 Center for Molecular Biomedicine, University of Medicine and Pharmacy at Ho Chi Minh City, Ho Chi Minh City, Vietnam; 3 Department of Pediatrics, University of Medicine and Pharmacy at Ho Chi Minh City, Ho Chi Minh City, Vietnam; 4 Department of Biomedical Science, Graduate School of Ajou University, Suwon, South Korea; 5 Translational Research Laboratory for Inflammatory Disease, Clinical Trial Center, Ajou University Medical Center, Suwon, South Korea

**Keywords:** Osteopontin, Transforming growth factor beta

## Abstract

Patients with late-onset asthma (LOA) have poor clinical outcomes. Osteopontin (OPN) is associated with airway inflammation and remodeling. To investigate the role of OPN in LOA compared to early-onset asthma (EOA), serum OPN levels were compared between 131 adult asthma patients (48 LOA and 83 EOA patients) and 226 healthy controls (HCs). BALB/c mice were sensitized with ovalbumin with/without polyinosinic-polycytidylic acid (poly(I:C)) from week 6 (A6 mice) or week 12 (A12 mice) after birth. Airway hyperresponsiveness (AHR), bronchoalveolar lavage fluid (BALF), cell counts, histology, and *Spp1* expression were assessed. The levels of OPN, transforming growth factor β1 (TGF-β1), chitinase 3-like 1 (CH3L1), and interleukin (IL) 5 were measured by ELISA. The expression of Smad3 phosphorylation and tissue transglutaminase 2 (TGM2) was evaluated by Western blot. The serum OPN levels were significantly higher in asthma patients than in HCs and in LOA patients than in those with EOA (*P* < 0.05) and were positively correlated with serum TGF-β1 and CH3L1 (*r* = 0.174, *r* = 0.264; *P* < 0.05). A12 mice showed elevated AHR with increased levels of OPN/TGF-β1/IL-5 in BALF and *Spp1* compared to A6 mice. Poly(I:C) induced remarkable TGF-β1, CH3L1, Th2 cytokine, and OPN levels in BALF and the expression of phosphorylated Smad3, TGM2, and *Spp1* in the lungs. OPN triggered TGF-β1/Smad3 signaling in the lungs, which was suppressed by dexamethasone and anti-IL5 antibody. In conclusion, aging and exposure to viral infections may induce OPN release and consequently modulate inflammation and TGF-β1/Smad3-related remodeling, contributing to the development of LOA.

## Asthma: Inflammatory protein drives adult-onset disease

Aging and viral infections in older individuals may combine to spur the release of an inflammatory protein implicated in late-onset asthma. A team led by Hae-Sim Park from Ajou University School of Medicine, Suwon, South Korea, showed that people who develop asthma after age 40 have higher blood levels of osteopontin, a multifunctional protein with roles in airway inflammation and tissue remodeling, than people who develop asthma at a younger age or healthy individuals. The researchers developed two ovalbumin-induced asthma models in younger and older mice, and found that older mice developed more severe airway hyperresponsiveness with higher levels of osteopontin, among other inflammatory markers, which were emnhanced by viral infection. Drug therapies that target osteopontin signaling could help combat the late-onset asthma.

## Introduction

The prevalence of asthma in the aged populations is increasing, causing an economic burden in Korea and worldwide. Asthma is characterized by chronic inflammation of the respiratory tract and consists of various phenotypes^1^. Late-onset asthma (LOA) is a phenotype that results in the development of asthma symptoms after 40 years of age and is characterized as being less eosinophilic, with a female predominance and more severe clinical features associated with a progressive decline in lung function^2,3^. Non-T helper (Th)2-LOA is usually corticosteroid-resistant and requires special treatment strategies^2^. Additionally, age-related changes in the immune system could complicate the pathophysiology of LOA^4,5^. The increasing prevalence and poor prognosis of LOA pose a challenge in the management of adult asthma. Attempts to elucidate the pathogenic mechanisms of LOA are warranted.

Airway remodeling consists of changes in the components and organization of the airway wall, which occurs in the epithelium, subepithelial layer, extracellular matrix (ECM) proteins, smooth muscle, and blood vessels^6^. The airway epithelium contributes to epithelial–mesenchymal transition, which leads to epithelial damage^7^. In subjects with LOA, airway remodeling can be either pathological or physiological due to aging-related structural changes in the airway^2,8^. Additionally, viral respiratory infections can worsen airway remodeling in adult asthma^9^. Respiratory viral infections induce the release of proinflammatory cytokines such as interleukin (IL)-6 and IL-8 from human airway epithelial cells (HAECs) and initiate cell recruitment, which amplifies airway inflammation and subsequent airway remodeling, such as long-term alterations in airway structures and functions in asthma^10,11^. A history of upper and lower respiratory infections, including acute bronchitis, the common cold and sinusitis, strongly increases the risk of asthma development in adults^12^. Furthermore, individuals with atopic asthma have been found to have an impaired response to viral infection^13^. Therefore, in addition to the aging process, respiratory viral infection may be an important risk factor that regulates airway remodeling in LOA.

Recently, osteopontin (OPN), a noncollagenous ECM protein, has been shown to be widely expressed in various cells, including HAECs, and is known as a multifunctional cytokine, as it drives Th1, Th2, and Th17 immunity^14–16^. OPN is known to act as a dual-regulatory cytokine—a proinflammatory cytokine during primary sensitization while having an anti-inflammatory effect on Th2 responses during a secondary antigen challenge^16^. More importantly, OPN is upregulated and remains a key component in allergen-induced airway remodeling in mouse models of asthma^17,18^. In patients with severe refractory asthma, OPN levels were shown to be associated with increased levels of transforming growth factor-β1 (TGF-β1)^19^. Epithelial OPN has been found to increase with IL-6/soluble IL-6 receptor stimulation in individuals with asthma^20^. Eosinophil-derived OPN was shown to contribute to fibrosis through the IL-33/amphiregulin (Areg)/epidermal growth factor receptor (EGFR) axis in the context of Th2 inflammation^21^. Recent functional studies demonstrated that OPN presents as the polymerized form due to the effect of tissue transglutaminase 2 (TGM2) in the human airways, which increases its collagen-binding activity^22,23^. Accumulating evidence suggests that OPN is a key cytokine in the modulation of inflammation and fibrotic events in asthmatic airways. Nevertheless, the role of OPN in the LOA phenotype has not yet been investigated. A subgroup of adults with asthma with high chitinase 3-like 1 (CH3L1, also called YKL-40) was prone to have adult-onset and frequent asthma exacerbations, suggesting an association between OPN and CH3L1^24^.

In the present study, we hypothesized that respiratory viral infection as well as aging could induce OPN release, thereby contributing to airway inflammation and remodeling in the pathogenesis of LOA. In addition, relationships with various inflammatory cytokines, including Th1/Th2 cytokines (IL-4, IL-5, IL-13, interferon [IFN]-γ) and epithelial and ECM cytokines (IL-8, IL-33, TGF-B1 and CH3L1), were evaluated in in vitro and in vivo models. Two different mouse models of asthma representing younger and older onset times were established. The effects of exposure to respiratory viruses on innate immunity and asthma severity were investigated using in vitro and in vivo models. Moreover, the downstream effects of OPN on fibrosis-associated pathways were explored.

## Materials and methods

### Study population

This study was approved by the Ethics Committee of Ajou University Institutional Review Board (AJIRB-GEN-SMP-13-108). In this study, 131 adults with asthma, including 48 with LOA and 83 with early onset-onset asthma (EOA), and 226 non-atopic healthy controls (HCs), were enrolled at the Department of Allergy and Clinical Immunology, Ajou University Hospital (Suwon, South Korea). The individuals with asthma were diagnosed and treated following the Global Initiative for Asthma (GINA) 2019 guideline^25^. Patients with primary eosinophilic diseases, including eosinophilic granulomatosis with polyangitis and hypereosinophilic syndrome, were excluded. We obtained written consent from each subject. The demographic data were recorded regarding age, sex, asthma onset time, asthma duration and smoking status. A diagnosis of atopy was based on positive skin test results for at least one or more common inhalant allergens (tree mixture, grass mixture, mugwort, ragweed, cat fur, dog fur, *Dermatophagoides farinae* (Der F*), Dermatophagoides pteronyssinus* (Der P) and *Alternaria* spp. [Bencard Co., Bredford, UK]). Patients with asthma underwent spirometry (FEV_1_%, FVC% predicted values) and methacholine (Mct) challenge tests to evaluate airway hyperresponsiveness (AHR) according to the European Respiratory Society standard^26^. The concentration of Mct required to produce a 20% decrease in FEV_1_ from baseline (MctPC20) was recorded. Severe asthma was defined according to the American Thoracic Society/European Respiratory Society guidelines^27^. Serum samples from patients and HCs were collected, stored at −70 °C and thawed before use. Total IgE levels in serum were measured by the ImmunoCAP system (Thermo Fisher Scientific, Waltham, MA, USA) in the detection range of 2–5000 kU/L.

### Classification of asthma phenotype

LOA and EOA were defined when asthma had been diagnosed at the age of ≥40 years and <40 years, respectively^28^. To identify eosinophilic asthma, we used blood eosinophil counts with the cutoff at 300 cells/µl as previously described^29^.

### HAEC cultures and treatment

HAECs, including A549 cells and primary small airway epithelial cells (SAECs), were purchased from the American Type Culture Collection (ATCC) (Manassas, VA, USA). A549 cells were cultured in RPMI-1640 medium supplemented with 10% fetal bovine serum, penicillin G sodium (100 UI/mL) and streptomycin sulfate (100 μg/mL) (all from Gibco, Grand Island, NY, USA). SAECs were cultured in basal medium supplemented with a bronchial epithelial cell growth kit (ATCC), penicillin G sodium (10 UI/mL), streptomycin sulfate (10 μg/mL) (Gibco), and amphotericin B (25 ng/mL) (Sigma Aldrich, St. Louis, MO, USA) according to the manufacturer’s protocol. Cells were grown at 37 °C in humidified air with 5% CO_2_. For treatment, cells (2 × 10^5^) were seeded onto a 12-well plate and stimulated with polyinosinic:polycytidylic acid (poly(I:C)) (Sigma Aldrich) at 1 and 10 μg/mL. After 24-h incubation, the supernatant was collected; cells were lysed in radioimmunoprecipitation assay (RIPA) buffer supplemented with protease inhibitor (Thermo Fisher Scientific) and stored at −70 °C for further experiments.

### Establishment of an LOA mouse model

Female BALB/c mice at 6 and 12 weeks old (weight 20 ± 2 and 21 ± 2 g, respectively) were purchased from the Jackson Laboratory (Bar Harbor, ME, USA), housed under specific pathogen-free conditions, maintained on a 12-h light/dark cycle and fed ad libitum. Asthma was induced at two time points, modified from a previous protocol^30^. Briefly, on days 0 and 14, mice were intraperitoneally sensitized with ovalbumin (OVA)/aluminum hydroxide (Alum) solution at 10 µg/1 mg. On days 28–30, the mice were challenged with 2% OVA for 30 min using an ultrasonic nebulizer (NE-SM1; Ktmed Inc., Seoul, South Korea). To establish the mouse model of virus-induced asthma exacerbation, mice were administered intranasal poly(I:C) (10 µg/mouse) prior to sensitization/challenge. To investigate the effects of OPN on asthma, mice were treated intranasally with 4 μg of mouse recombinant OPN protein (rOPN, 763606, Biolegend, San Diego, CA, USA) for 1 h prior to sensitization on days 0 and 14. In some experiments, mice were given dexamethasone 21-phosphate disodium salt (D1159) (Dex, 1 mg/kg), montelukast sodium hydrate (Mon, 10 mg/kg) or anti-IL-5 antibody (504302) (anti-IL5, 20 mg/kg) for 3 consecutive days prior to the challenge. Mice were assayed at 24 h after the last challenge. All animal experiments were approved by the Institutional Animal Care and Use Committee of Ajou University (IACUC 2018-0041). OVA, Dex and Mon were from Sigma Aldrich, Alum was from Thermo Fisher Scientific, and the anti-IL-5 antibody was from Biolegend.

### Measurement of AHR

AHR to acetyl-β-methylcholine chloride was recorded using the FlexiVent system (Scireq, Montreal, QC, Canada). Mice were anesthetized with pentobarbital sodium, intubated with a cannula and ventilated with a tidal volume of 10 mL/kg at a frequency of 150 breaths/min. The airway resistance (*R*_L_) at baseline and a dilution series of MC from 1.56 to 25 mg/mL were noted.

### Analysis of differential cell count and histology

To collect bronchoalveolar lavage fluid (BALF), 1 mL of 1x phosphate buffered saline (PBS) plus 1% bovine serum albumin (Sigma Aldrich) was used to wash the trachea. The BALF was centrifuged at 1200 revolutions per minute for 5 min at 4 °C, and the supernatant was harvested and stored at −70 °C until further experiments. Total and differential cell counts were determined by using a hemocytometer and hematoxylin–eosin (HE)-stained slides as previously described^30^.

Consecutively, lung tissues were perfused and divided in half; the left parts were fixed in 4% paraformaldehyde, while the right lung tissues were divided for the isolation of RNA (immersed in RNAlater® solution, Thermo Fisher Scientific) and proteins. The fixed tissues were embedded in paraffin and sectioned at 5-μm thickness. To visualize infiltrated cells, mucus-containing cells, and collagen deposition, tissues were stained with HE, periodic acid-Schiff, and Masson’s trichrome, respectively. The number of cells in the peribronchial, perivascular, and mucus-secreting cell areas, as well as smooth muscle thickness and collagen deposition were counted.

### Measurement of inflammatory cytokines

The levels of inflammatory cytokines in the serum, cell-free supernatant and BALF were measured by enzyme-linked immunosorbent assay (ELISA) according to the manufacturer’s protocol. ELISA kits for human TGF-β1/CH3L1/OPN, mouse TGF-β1/IL-33 and CH3L1 were purchased from R&D Systems, Inc. (Minneapolis, MN, USA). The human IL-8 ELISA kit was purchased from Endogen (Woburn, MA, USA). For mouse BALF, the ELISA kits used to measure the levels of mouse OPN and eotaxin-2 were purchased from Ray Biotech, Inc. (Norcross, GA, USA), and mouse IL-4, IL-5, IL-13 and IFN-γ were purchased from Thermo Fisher Scientific.

### Immunohistochemistry

Briefly, tissue antigen retrieval was performed with sodium citrate buffer (pH 6.0), followed by incubation with blocking buffer (0.05% PBS-Tween 20 containing 5% bovine serum albumin and 10% normal donkey serum) for 1 h at room temperature. Tissues were then incubated with rabbit anti-mouse TGM2 antibody (3357S, Cell Signaling Technology, Inc., Danvers, MA, USA) at 4 °C overnight in a humidified chamber. Subsequently, tissues were incubated with Alexa Flour 488-conjugated donkey anti-rabbit antibody (Thermo Fisher Scientific) for 1 h at room temperature, followed by counterstaining with 4',6-diamidino-2-phenylindole (1 μg/mL) for 10 min and finally mounting with a mounting solution (Biodema Corp, Foster City, CA, USA). All reagents were purchased from Sigma Aldrich unless indicated otherwise.

### Analysis of gene expression by real-time PCR

Lung tissues were homogenized and harvested for total RNA using TRIzol® (Thermo Fisher Scientific). Total RNA was synthesized for cDNA by the SuperScript^TM^ First Strand Synthesis System (Thermo Fisher Scientific). Gene expression analysis using cDNA as templates was conducted using the KAPA SYBR® FAST qPCR Master Mix (2x) kit (Boston, MA, USA). The sequences of the primers are displayed in Table S1. The annealing temperature was 60.6 °C for all genes.

### Detection of Smad3 intermediate signaling by Western blotting

Proteins were isolated from the right lung tissues by lung homogenate, followed by incubation with RIPA buffer. Aliquots of 35 μg of protein were loaded onto 10% sodium dodecyl sulfate polyacrylamide gels and transferred to polyvinylidene difluoride membranes (Bio-Rad, Hercules, CA, USA). After blocking in 5% skim milk (Sigma Aldrich) in Tris buffered saline containing 0.05% Tween 20 (TBS-T) for 1 at room temperature, membranes were incubated with primary antibodies against OPN (R&D Systems), Smad3, phosphorylated Smad3 (pSmad3) (Abcam, Cambridge, UK), and TGM2 overnight at 4 °C with gentle shaking. Then, membranes were washed three times with TBS-T for 10 min each and incubated with horseradish peroxidase conjugated with anti-goat or anti-rabbit antibody for 1 h at room temperature. Anti-β-actin antibody (Thermo Fisher Scientific) was used as an internal control. Signals were detected using ECL Plus Western Blotting Detection Reagents (GE Healthcare, Little Chalfont, UK). The intensity of bands was analyzed using a gel doc system (Bio-Rad Laboratories, Inc., Hercules, CA, USA).

### Statistical analysis

The normality test of data was checked with the Kolmogorov–Smirnov test. Comparisons between the two groups were performed by the Mann–Whitney *U* test for continuous variables and Pearson’s chi-squared test for categorical variables. Bivariate correlations were analyzed with Spearman's rank correlation coefficient test and are presented as scatter plots. Multiple comparisons of data from multiple groups were approached by one-way ANOVA with the Tukey post hoc test. A receiver operating characteristic (ROC) curve analysis was applied to estimate the diagnostic values of serum cytokines. Data analysis and graph preparation were performed by using the statistical software packages IBM SPSS 20.0 (Armonk, NY, USA) and GraphPad Prism 6 (San Diego, CA, USA), respectively. Heatmap graphs were drawn by R (R Core Team 8, Vienna, Austria). Significance levels for all analyses were set at *P* < 0.05.

## Results

### Clinical characteristics of the study subjects

The demographic characteristics of the study subjects enrolled are summarized in Table 1. Subjects with asthma were older than the HCs (*P* < 0.001), and there were more females in the asthma group than in the HC group (*P* = 0.023). Compared to patients with EOA, those with LOA were older and less atopic (*P* < 0.001 and *P* < 0.001, respectively). There were no significant differences in peripheral eosinophil counts, FEV_1_% predicted values or serum total IgE levels between the two phenotypes. Subjects with asthma showed significantly higher serum levels of IL-8, CH3L1, and TGF-β1 than HCs (all *P* < 0.01). The serum levels of CH3L1 remained significantly increased in patients with LOA compared to those with EOA (*P* < 0.05).

**Table 1:** Demographic characteristics and serum cytokine quantifications.

	Asthma (*n* = 131)	HC (*n* = 226)	*P-*value	LOA (*n* = 48)	EOA (*n* = 83)	*P*-value
Age (years)	43.61 ± 13.35	31.43 ± 9.95	<0.001	54.81 ± 8.63	37.13 ± 11.12	<0.001*
Female (*n*, %)	83/131 (63.36%)	101/223 (45.29%)	0.023	27/48 (56.63%)	56/83 (67.47%)	0.002
Atopy (*n*, %)	70/131 (53.44%)	52/188 (27.66%)	<0.001	23/48 (47.92%)	47/83 (56.62%)	<0.001
TEC (count/μL)	534.91 ± 1540.68	NA	NA	770.92 ± 2459.24	391.51 ± 367.54	0.381*
FEV_1_ (%) predicted	88.05 ± 18.70	NA	NA	89.0 ± 21.81	87.64 ± 17.43	0.783
FVC (%) predicted	91.84 ± 19.27	NA	NA	96.45 ± 16.20	89.81 ± 20.32	0.214
FEV_1_/FVC ratio	0.95 ± 0.09	NA	NA	0.94 ± 0.11	0.95 ± 0.09	0.815
MctPC20 (mg/mL)	9.29 ± 15.79	NA	NA	12.89 ± 19.62	7.31 ± 12.96	0.106
log total IgE (kU/L)	2.35 ± 0.55	1.78 ± 0.67	<0.001	2.39 ± 0.62	2.32 ± 0.5	0.734*
Eos asthma	47/110 (42.73%)	NA	NA	19/42 (45.24%)	28/68 (41.18%)	0.412
log OPN (pg/mL)	3.41 ± 0.54	2.99 ± 0.83	<0.001*	3.57 ± 0.41	3.31 ± 0.59	0.002*
log IL-8 (pg/mL)	1.16 ± 0.40	1.46 ± 0.60	0.001*	1.22 ± 0.45	1.13 ± 0.37	0.427*
log TGF-β1 (ng/mL)	1.45 ± 0.24	1.35 ± 0.23	0.002	1.46 ± 0.25	1.44 ± 0.23	0.419*
log CH3L1 (pg/mL)	1.65 ± 0.31	1.53 ± 0.24	0.003*	1.79 ± 0.32	1.55 ± 0.26	<0.001*

Categorical values are presented as percentages and analyzed by Pearson’s chi-squared test. Numerical values are presented as the means ± SD and analyzed by Student’s *t* test or (*) the Mann–Whitney *U* test. Log: skewed data were log-transformed.

*CH3L1* chitinase-3-like-1, *IL* interleukin, *FVC* forced vital capacity, *FEV*_*1*_ forced expiratory volume in 1 s, *MctPC20* the concentration of methacholine to produce a 20% decrease in FEV_1_, *OPN* osteopontin, *TEC* total eosinophil count, *TGF-β1* transforming growth factor β1.

### Increased levels of serum OPN with age

**Fig. 1:**
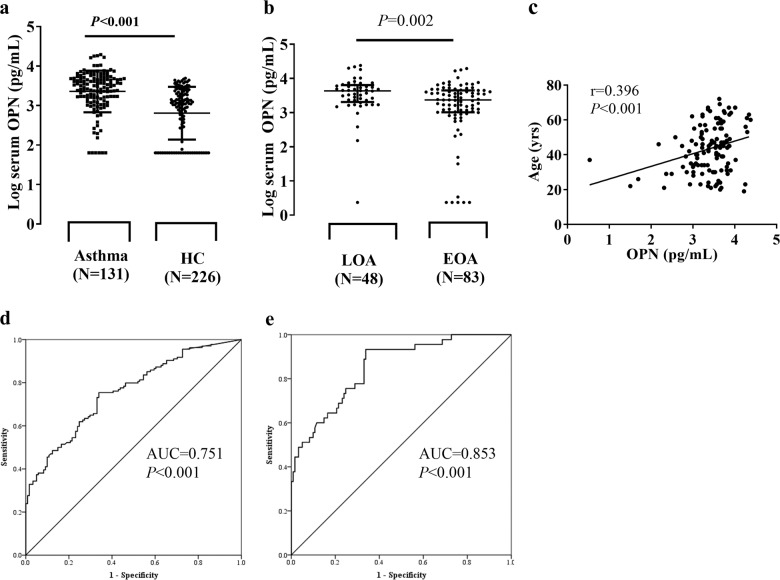
Increased serum OPN levels with age in individuals with asthma. Comparison between **a** patients with asthma vs. HCs and **b** patients with LOA vs. those with EOA. *P* values were analyzed by the Mann–Whitney *U* test. **P < 0.01, ***P < 0.001 between the groups. Data are shown as log-transformed, mean ± SEM. **c** Correlation between serum OPN and age, analyzed by Spearman’s rank correlation coefficient. Diagnostic values of serum OPN level in the discrimination of individuals with asthma **d** and individuals with LOA **e** from HCs. AUC area under the curve; EOA early-onset asthma, HCs healthy controls, LOA late-onset asthma, OPN osteopontin, ROC receiver operating characteristic.

The serum levels of OPN were significantly higher in subjects with asthma than in HCs (3.41 ± 0.54 vs. 2.99 ± 0.83 pg/mL) (Fig. 1a) and in patients with LOA compared to those with EOA (3.57 ± 0.41 vs. 3.31 ± 0.59 pg/mL) (Fig. 1b and Table 1). The serum OPN levels in subjects with asthma were positively correlated with age (*r* = 0.396, *P* < 0.001) (Fig. 1c).

### Diagnostic values of OPN in the discrimination of individuals with asthma and patients with LOA from HCs

The ROC curve analysis of serum OPN was performed for differentiating individuals with asthma from HCs with an area under the curve (AUC) value of 0.757 (*P* < 0.001) and an optimal cutoff of 1488.51 pg/mL (log-transformed data as 3.17) at 75.4% sensitivity and 66.1% specificity (Fig. 1d). Based on the given cutoff value, subjects were divided into high-OPN and low-OPN responders. Additionally, the serum OPN levels were significantly different between patients with LOA and HCs, with an AUC value of 0.853 (*P* < 0.001) at 93.3% sensitivity and 66.1% sensitivity (Fig. 1e).

### Association of serum OPN with clinical characteristics and other inflammatory cytokines

Differences in clinical characteristics between high-OPN and low-OPN responders are displayed in Table 2. High-OPN responders were older and had a higher prevalence of atopy than low-OPN responders (both *P* < 0.05). There were no significant differences between high-OPN and low-OPN responders in sex, total IgE level, and baseline values of FEV_1_/FVC and MctPC20. For inflammatory cytokines, high-OPN responders displayed significantly higher serum levels of IL-8, CH3L1, and TGF-β1 (all *P* < 0.05).

**Table 2:** Comparison of demographic characteristics between high-OPN and low-OPN responders.

	High-OPN responders (*n* = 142)	Low-OPN responders (*n* = 113)	*P* values
Age (years)	40 ± 14.00	30 ± 10.00	<0.001*
Female (*n*, %)	75/142 (52.82%)	63/113 (55.75%)	0.367
Atopy (*n*, %)	55/138 (39.85%)	17/86 (19.77%)	0.002
TEC (count/μL)	570.77 ± 1754.63	430.45 ± 362.1	0.387*
FEV_1_% predicted	87.39 ± 17.59	89.68 ± 21.62	0.155
FVC% predicted	89.61 ± 17.77	97.31 ± 22.12	0.647
FEV_1_/FVC ratio	0.96 ± 0.1	0.93 ± 0.07	0.317
MctPC20 (mg/mL)	9.19 ± 17.1	9.22 ± 11.24	0.309
log total IgE (kU/L)	2.22 ± 0. 61	2 ± 0.71	0.076
Severe asthma (*n*, %)	19/101 (18.81%)	9/33 (27.27%)	0.328
Eos asthma (*n*, %)	40/84 (47.62%)	15/29 (51.72%)	0.83
log IL-8 (pg/mL)	1.70 ± 2.25	1.28 ± 1.75	<0.001*
CH3L1 (ng/mL)	59.75 ± 57.6	42.29 ± 27.6	0.008
TGF-β1 (ng/mL)	30.31 ± 15.66	25.04 ± 13.58	0.026

Categorical values are presented as percentages and analyzed by Pearson’s chi-squared test. Numerical values are presented as the means ± SD and analyzed by Student’s *t* test or (*) the Mann–Whitney *U* test. Log: skewed data were log-transformed.

*CH3L1* chitinase-3-like-1, *IL* interleukin, *FVC* forced vital capacity, *FEV*_*1*_ forced expiratory volume in 1 second, *MctPC20* the concentration of methacholine to produce a 20% decrease in FEV_1_, *OPN* osteopontin; *TEC* total eosinophil count, *TGF-β1* transforming growth factor β1.

Significantly positive correlations were noted between serum OPN and IL-8/TGF-β1/CH3L1 (*r* = 0.282, *r* = 0.174, *r* = 0.264; all *P* < 0.05), while a negative correlation between OPN and eotaxin-2 was noted (*r* = −0.337, *P* < 0.05) (data not shown).

### Characteristics of LOA and EOA mouse models

**Fig. 2:**
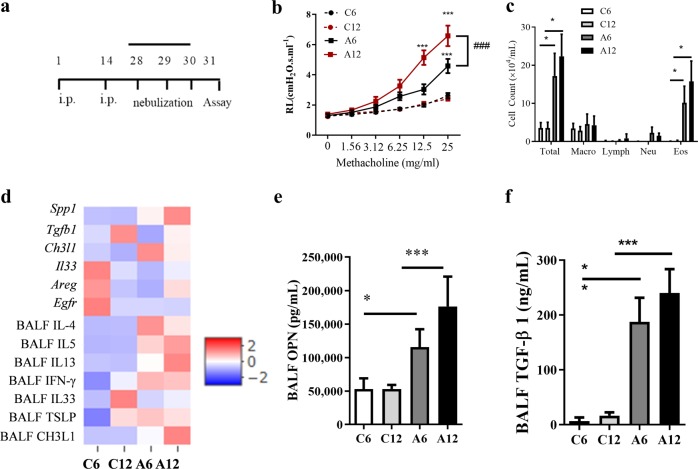
Increased airway inflammation and AHR in older and younger mouse models of asthma. **a** Mice at 6 and 12 weeks old underwent the induction of asthma by using OVA/Alum and were assessed for the severity of asthma according to the described protocol. Mice were assessed for **b** AHR to methacholine at different concentrations (0, 1.56, 3.12, 6.25, 12.5, and 25 mg/mL). ***P < 0.001 at the respective weeks, between the negative control group (C6, C12) *vs* the asthma group (A6, A12); ^###^*P* < 0.001 between the asthma group at 6 and 12 weeks. **c** Total and eosinophil counts in BALF. Mouse lung tissues were used to analyze gene expression, while BALF was measured for inflammatory cytokines. **d** Heatmap clustering was applied to visualize the relative changes in gene expression and levels of proinflammatory cytokines according to onset age. BALF levels of **e** OPN and **f** TGF-β1 were elevated with onset age. Data are presented as the means ± SEM, *n* = 10 for each group. *P < 0.05, **P < 0.01, ***P < 0.001 between the groups. AHR airway hyperresponsiveness; Alum aluminum hydroxide; Areg amphiregulin; BALF bronchoalveolar lavage fluid; CH3L1 chitinase 3-like 1; Dex dexamethasone; Egfr epidermal growth factor receptor; Eos eosinophil; IFN-γ interferon γ; IL interleukin; Lymph lymphocyte; Macro macrophage; Mon montelukast; Neu neutrophil; OPN osteopontin; poly(I:C) polyinosinic:polycytidylic acid; Spp1 secreted phosphoprotein 1; Total total cell count; TGF-β1 transforming growth factor β1; TSLP thymic stromal lymphopoietin.

The induction of asthma led to significantly higher AHR and total cell/eosinophil counts in the BALF of older mice at 12 weeks after birth (A12 mice) than in younger mice with asthma at 6 weeks after birth (A6 mice) (Fig. 2a–c). Changes in Th2 cytokines were observed as decreased levels of IL-4 but increased levels of IL-5 and IL-13 (Fig. 2d). Genes associated with ECM remodeling and fibrosis (*Spp1*, *Areg, Egfr, Tgfβ1, Ch3ll*) and epithelial inflammation (*IL33*) were differentially expressed in A12 mice. *Spp1* was upregulated, and the levels of OPN and TGF-β1 were increased in BALF (A6 vs. A12 mice, *P* < 0.05 for all, Fig. 2d–f). Histological analysis revealed a tendency toward an increase in infiltrated cells, mucus secretion, and collagen deposition (Figs. S2 and S3). These findings led us to investigate whether exogenous factors, including environmental factors, may worsen asthma severity.

### Effect of respiratory virus on airway inflammation in mouse models of asthma: comparison between younger and older mice with asthma

**Fig. 3:**
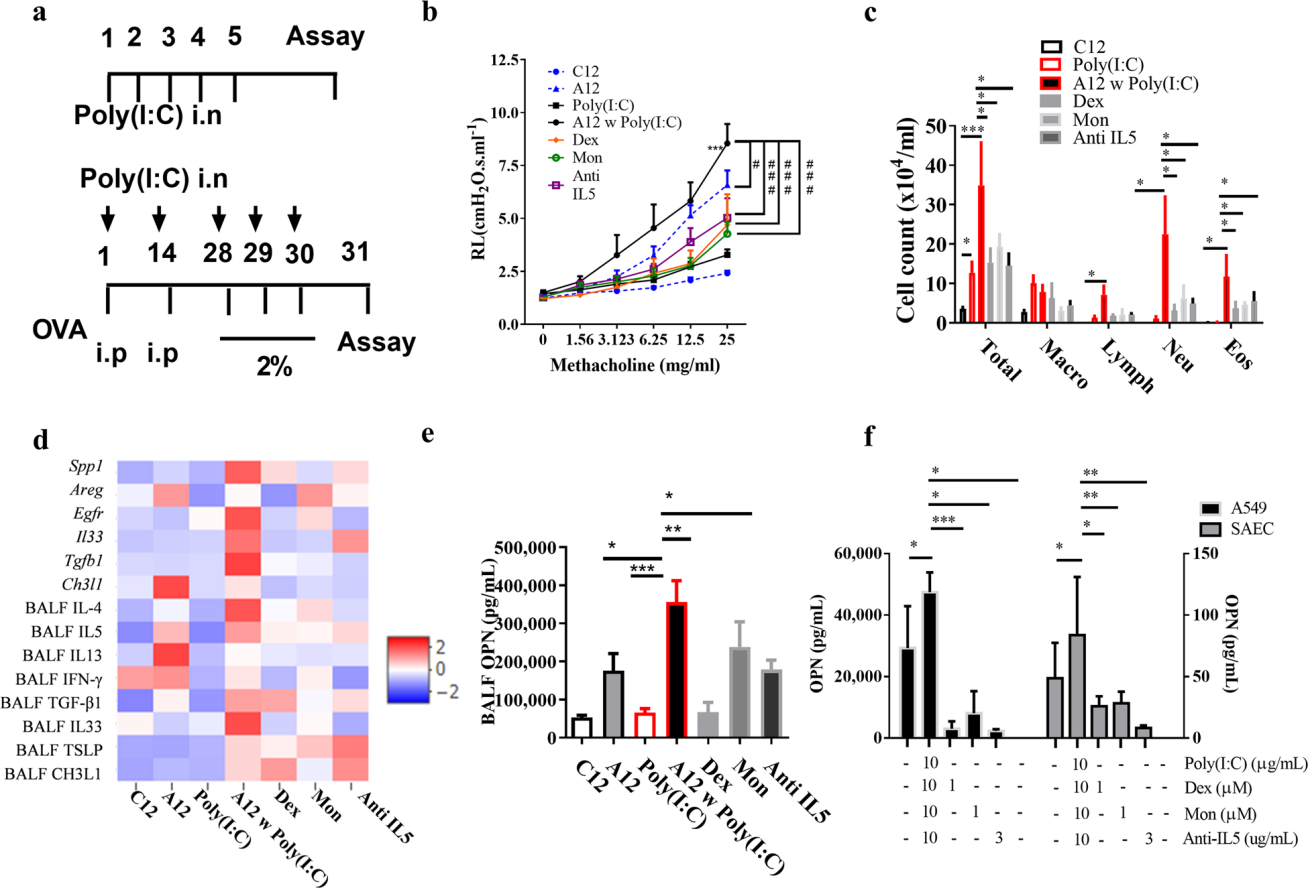
Virus-induced asthma exacerbation in older mice. **a** Intranasal poly(I:C) 10 μg was administered to mice either for 5 days or prior to OVA sensitization/challenge as in the described protocol. **b** AHR to methacholine was measured on the indicated days. Some mice were treated with dexamethasone (Dex) 1 mg/kg, montelukast (Mon) 10 mg/kg, or anti-IL5 antibody (20 μg per mouse) for 3 days prior to OVA challenge. ***P < 0.001 vs. the control, ^#^*P* < 0.05, ^###^*P* < 0.001 between the A12 w Poly(I:C) group and drug-treated mice. **c** Effects of virus and drugs on total cell, neutrophil, and eosinophil counts in BALF. *P < 0.05, ***P < 0.001 between the groups; NS not significant. **d** Relative changes in gene expression and levels of proinflammatory cytokines in BALF. **e** The levels of OPN in BALF increased with viral exposure and decreased with anti-asthmatic drugs. *P < 0.05, **P < 0.01, ***P < 0.001 between the indicated groups. In an in vitro experiment, HAECs were stimulated with poly(I:C) at 1 and 10 μg/mL and treated with Dex, Mon, and anti-IL5 antibody at the indicated concentrations. **f** Levels of OPN in the cell culture supernatants were measured by ELISA. *P < 0.05, **P < 0.01, ***P < 0.001 between the groups. AHR airway hyperresponsiveness; Alum aluminum hydroxide; Areg amphiregulin; BALF bronchoalveolar lavage fluid; CH3L1 chitinase 3-like 1; Dex dexamethasone; Egfr epidermal growth factor receptor; Eos eosinophil; IFN-γ interferon γ; IL interleukin; Lymph lymphocyte; Macro macrophage; Mon montelukast; Neu neutrophil; OPN osteopontin; poly(I:C) polyinosinic:polycytidylic acid; Spp1 secreted phosphoprotein 1; Total total cell count; TGF-β1 transforming growth factor β1; TSLP thymic stromal lymphopoietin.

Given that respiratory viruses, especially RNA viruses such as rhinovirus, can cause asthma exacerbation, we administered poly(I:C) to C12 (Poly(I:C)) and A12 (A12 w Poly(I:C)) mice (Fig. 3a). Treatment with poly(I:C) alone increased AHR, total cell counts and OPN levels, although there were no significant differences between the groups. The administration of poly(I:C) to A12 [A12 w Poly(I:C)] mice robustly aggravated AHR and induced mixed cellular (including neutrophils and eosinophils) infiltration in BALF (Fig. 3b, c, *P* < 0.05 for all). In the A12 w Poly(I:C) group, poly(I:C) enhanced the expression of most of the genes investigated (*Spp1*, *Egfr*, *Il33, Tgfb1*) as well as Th2 cytokines (IL-4, IL-5, IL-13) and epithelial cell/ECM-derived cytokines such as OPN, TGF-β1, IL-33, TSLP, and CH3L1 (Fig. 3d, e). In histological examinations, A12 w Poly(I:C) strongly enhanced inflammatory cell infiltration and mucus secretion as well as collagen deposition (Figs. S2 and S3).

### Regulatory function of OPN on TGF-β1/Smad3 signaling pathways

**Fig. 4:**
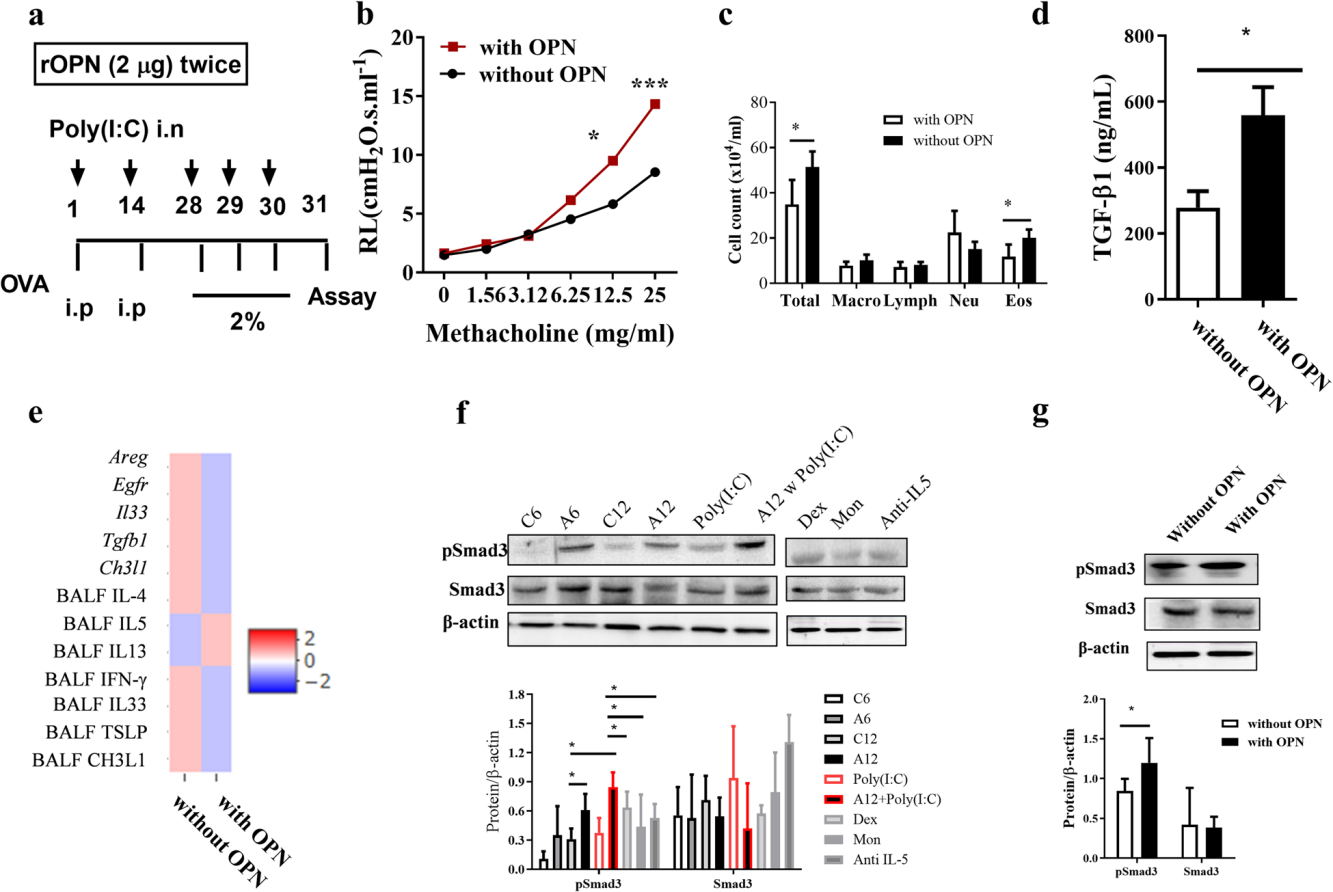
Effects of OPN on the TGF-β1/Smad3 signaling pathway. **a** In total, 4 μg of recombinant OPN (rOPN) was administered to mice during the sensitization period as in the described protocol. Increased **b** AHR and **c** mixed eosinophil/neutrophil counts in BALF. **d** Increased TGF-β1 levels in BALF after rOPN administration. **e** Changes in gene expression and cytokine levels in BALF. **f** and **g** Increased levels of pSmad3 in the lung tissues of mice, analyzed by Western blotting. Data are shown as the means ± SEM, *n* = 5–10 for each group. *P values were analyzed by the Mann–Whitney *U* test. AHR airway hyperresponsiveness; Alum aluminum hydroxide; Areg amphiregulin; BALF bronchoalveolar lavage fluid; CH3L1 chitinase 3-like 1; Dex dexamethasone; Egfr epidermal growth factor receptor; Eos eosinophil; IFN-γ interferon γ; IL interleukin; Lymph lymphocyte; Macro macrophage; Mon montelukast; Neu neutrophil; OPN osteopontin; poly(I:C) polyinosinic:polycytidylic acid; Total total cell count; TGF-β1 transforming growth factor β1; TSLP thymic stromal lymphopoietin.

The simultaneous increase in OPN and TGF-β1 raised the question of whether OPN has any regulatory effect on TGF-β1 secretion. The administration of rOPN to the A12 w Poly(I:C) group (w OPN) during the sensitization period significantly increased AHR compared to the nontreated group (without OPN) (Fig. 4a, b, *P* < 0.05); however, neutrophil/eosinophil counts were elevated in the BALF of the two groups, but without any statistical significance (Fig. 4c). There were no significant changes in the levels of inflammatory genes/cytokines, except for *Tgfb1* expression, but the TGF-β1 levels in BALF were significantly higher in the OPN-treated group than in the nontreated group (*P* < 0.05) (Fig. 4d). Interestingly, the phosphorylation of Smad3 was upregulated with age (A12 vs. A6 mice) in the A12 w Poly(I:C) group (*P* < 0.05, Fig. 4e, f). TGF-β1 gene expression, release, and activity were enhanced as OPN levels increased.

### Effects of anti-asthmatic drugs on OPN regulation

Mice were treated with Dex, Mon, and anti-IL5 antibody to evaluate whether these anti-asthmatic medications and type 2 biologics may downregulate OPN levels. Dex, Mon, and anti-IL5 antibody significantly suppressed AHR and neutrophil/eosinophil counts in BALF (Fig. 3b, c). Dex and anti-IL5 antibody were effective in attenuating *Spp1*, OPN, and TGF-β1 in BALF, while Mon was effective in reducing the levels of CH3L1 in BALF and *Ch3l1* expression (Fig. 3d, e). In an in vitro assay, exposure to Poly(I:C) released OPN, TGF-β1, and IL-8 from HAECs and CH3L1 from SAECs. Dex, Mon, and anti-IL5 antibody significantly suppressed epithelial-cell-derived OPN release (Figs. 3f and S1).

### Viral infection increases polymerization of OPN by TGM2 in A12

**Fig. 5:**
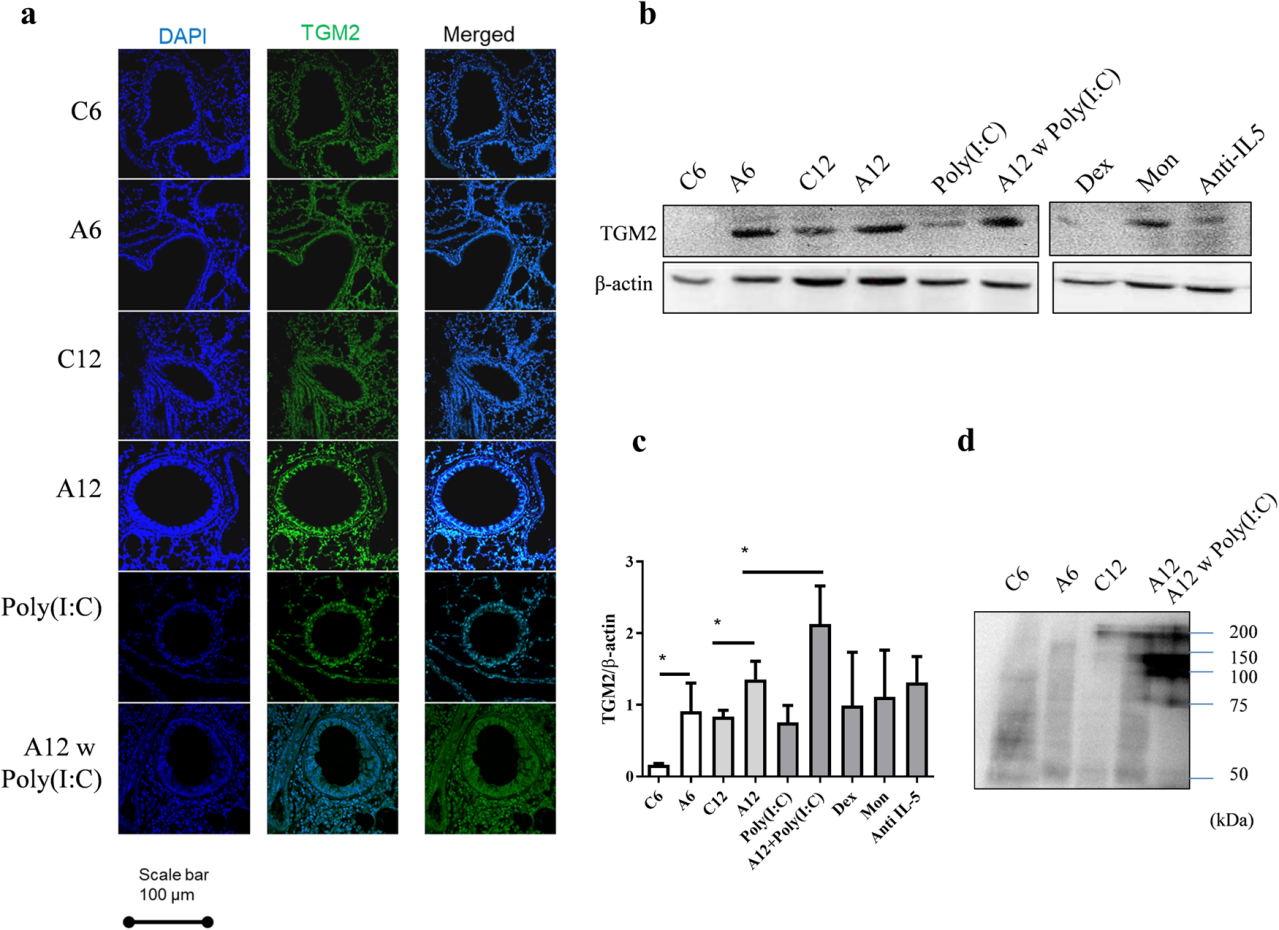
Increase in polymerized OPN and TGM2 expression with age and viral infection. The expression of TGM2 in the lung tissues of mice were analyzed using **a** immunohistochemistry and **b** & **c** Western blotting. **d** Increased polymerized OPN in mouse BALF as analyzed by Western blotting. *P < 0.05 compared between the indicated groups. Da daltons; OPN osteopontin; TGM2 tissue transglutaminase 2.

To elucidate the association between OPN and epithelial TGM2, the expression levels of TGM2 were compared in the lung tissues of mice with age and virus infection status. The A12 w Poly(I:C) group showed the highest expression of TGM2, which was highly localized in the epithelial layer and the peribronchial/perivascular area compared to the three other groups (*P* < 0.05) (Fig. 5a–c). TGM2 is known to polymerize OPN^22^, as shown in Fig. S3. Interestingly, in mouse BALF, Western blot analysis revealed the polymerized form of OPN with bands ranging from >55 to 200 kDa in the A6, A12, and A12 w Poly(I:C) groups (Fig. 5d). All anti-asthmatic drugs used in this study could not significantly suppress the increased TGM2 level.

## Discussion

This study demonstrated the role of OPN as a central mediator in the pathogenesis of LOA rather than EOA. The upregulation of OPN may be attributed to the aging process and viral infections in older subjects with asthma, which can accelerate airway remodeling by enhancing TGF-β1/Smad3-related pathways in the pathogenesis of LOA. In the milieu of aging and viral exposure, the structure of OPN was modified due to increased TGM2, subsequently enhancing fibrosis. This is the first investigation identifying higher serum OPN levels in patients with LOA and revealing the role of OPN using in vitro and in vivo models of LOA. LOA is considered a distinct phenotype of adult asthma presenting less eosinophilia and more severe clinical features with poorer prognosis than EOA^2,4^. Until now, serum CH3L1 and eotaxin-2 have been found to be upregulated in patients with LOA, although the age cutoff for LOA was variable among studies^24,31,32^. The present study demonstrated that the serum level of OPN among screened cytokines was upregulated in patients with LOA compared to those with EOA. Serum OPN level was found to have the potential to discriminate patients with LOA and those with adult asthma from HCs at 93.3% sensitivity. Several studies supported higher serum and sputum OPN levels in adults with asthma^19,33^. Bronchial OPN levels were correlated with impaired lung function in patients with severe refractory asthma, while sputum OPN levels were associated with the degree of neutrophilic inflammation^33–35^. The present study demonstrated a significant association between serum OPN and IL-8 levels in adults with asthma. IL-8 is a potent mediator inducing neutrophil migration and activation, which can additionally recruit eosinophils^36^. Thus, increased serum OPN levels have been shown to induce mixed neutrophilic/eosinophilic inflammation in adults with asthma. Serum OPN may be a potential, noninvasive biomarker for the early diagnosis of LOA in adults.

Attempts have been made to elucidate the mechanism underlying airway remodeling in LOA. In addition to environmental exposure, aging-related changes in airway structure and morphology may impact lung function and fibrosis in asthmatic airways^2,37^. Age at the initial time of allergen sensitization is critical for dictating the response upon subsequent exposure, as older mice showed increased expression of a steroid-resistant cascade of wingless/integrase and TGM2, which aggravated airway inflammation and remodeling^38,39^. OPN is known as a major ECM-inducing cytokine that modulates collagen deposition and fibrosis in asthma^17,21,33^. In this study, older mice with asthma showed increased airway inflammation and AHR compared to younger mice with differential expression of cytokines at the genetic and protein levels. T cell and innate immunity were upregulated by *Spp1/*OPN, Th2 cytokines (IL-5, IL-13), and ECM cytokines (TGF-β1 and CH3L1), while IL-4, IFN-γ, and epithelial cytokines (IL-33, TSLP) were downregulated. These findings were in partial agreement with those of previous studies using older mice with asthma^40,41^. Moreover, exposure to viral dsRNA poly(I:C) worsened AHR and induced mixed eosinophil/neutrophil infiltration in older mice with asthma, which might resemble the phenotype of LOA. Robust changes in gene/cytokine profiles were induced through the upregulation of OPN/*Spp1* and inflammatory cytokines. In addition, the administration of rOPN during sensitization greatly amplified AHR, inflammatory cells, and fibrosis in our virus-infected asthma mouse model. Interestingly, compared with younger mice with asthma, older mice with asthma displayed a higher expression of pSmad3^42^, an important pathway of TGF-β1-mediated fibrosis^43^, which was also strongly enhanced by poly(I:C). Chronic poly(I:C) stimulation can stimulate NF-κB expression and epithelial–mesenchymal transition, fibrosis, and airway remodeling^44^. Taken together, these findings suggest that viral exposure could amplify the age-dependent increase in OPN, which enables airway inflammation and TGF-β1-mediated remodeling and contributes to presenting a more severe phenotype of asthma of LOA.

Associations of serum OPN with TGF-β1 and CH3L1 led us to further investigate the role of OPN in the regulation of these ECM cytokines. Increasing evidence suggests that both TGF-β1 and CH3L1 are crucial for modulating airway remodeling and fibrosis in severe asthma^19,24,45,46^. In this study, viral exposure triggered direct activation of HAECs by the release of OPN, TGF-β1, and CH3L1 (mostly from primary small airway epithelial cells). Exposure to rOPN could induce the expression of *Tgfβ1* and the release of TGF-β1, IL-5, IL-13, and phosphorylated Smad3 in a mouse model of virus-induced asthma exacerbation. The latter was in line with the results of a previous study that showed that OPN-deficient mice had reduced TGF-β1^18^. TGF-β1 produces OPN through transcriptional regulation in osteosarcoma cells^47^. These findings show the bidirectional effects between OPN and TGF-β1 and suggest that OPN can trigger TGF-β1/Smad3-induced fibrosis in the airway of LOA. Regarding CH3L1, there were no observed causal relationships between OPN and CH3L1 in this context. However, TGF-β1 could stimulate chitinase-like 2, the closest homolog to CH3L1, in cultured macrophages^48^. Conversely, YKL-40 was found to induce TGF-β1 gene expression in fibroblasts^49^. Thus, we hypothesized that increased OPN enhances TGF-β1/Smad3-dependent fibrosis; consequently, TGF-β1 modulates CH3L1, which contributes to amplifying TGF-β1 expression. Further studies on the TGF-β1/CH3L1 axis are warranted.

The multiple functions of OPN have been extensively studied in various kinds of inflammation mechanisms. In addition to TGF-β1-dependent fibrosis, two other fibrosis-related downstream effects of OPN have been demonstrated in asthmatic airways. The polymerization of OPN leads to increased collagen binding under the effect of TGM2^22^. The latter has been found to be upregulated with aging^38^. OPN could be released from eosinophils reprogrammed by the Areg/IL33/EGFR pathway and initiated fibrosis^21^. In the present study, we could not detect any changes in Areg/IL33/EGFR. Instead, we found that TGM2 was highly upregulated by virus infection in older mice with asthma. Concomitantly, polymerized forms of OPN (similar to the structure of OPN, which was polymerized by TGM2) were detected in mice with asthma and enhanced after exposure to viruses. In addition, the polymerized form of OPN was found in asthmatic airways, while fragmented forms were found in the airway secretion of patients with asthma^23^. Although we could not find a direct effect of OPN on TGM2 formation, the current findings led us to speculate that viral infection and aging may stimulate TGM2 polymerization, which further enhances collagen binding. Moreover, an age-dependent increase in TGM2 has been reported to facilitate steroid insensitivity^38^. Thus, novel therapies targeting the TGM2/OPN axis could help prevent the development of steroid insensitivity and airway remodeling in LOA.

Finally, we aimed to investigate the potential therapies for the modulation of OPN in LOA, and, on the basis of our findings, we suggest that Dex and anti-IL5 antibody are potential drugs to suppress OPN-mediated airway inflammation and remodeling in the older mouse model of virus-induced asthma exacerbation. Regarding the effects of glucocorticoids on OPN release, there are some conflicting results showing that steroids increased OPN expression in cardiac myocytes and microvascular endothelial cells, while Dex suppressed OPN expression in a mouse model of allergic asthma^50,51^. Glucocorticoid receptors recognize an element on the promoter sequence of the OPN gene; thus, glucocorticoids may exert various responsiveness to the OPN promoter^52^. The increased level of OPN in LOA can be a potential target of glucocorticoid treatment. With respect to the association of OPN with IL-5, it has been suggested that OPN suppresses plasmacytoid dendritic cells, resulting in enhanced Th2 cytokines^16^. IL-5 induces eosinophil-derived OPN, which leads to angiogenesis in asthma^53^. Reslizumab, an anti-IL5 antibody, is effective in treating LOA patients with increased eosinophilia^54^. Our findings help elucidate mechanisms by which anti-IL5 antibody could effectively modulate the elevated OPN in the LOA phenotype. Therefore, Dex and anti-IL5 antibody could be beneficial in the treatment of LOA.

There is a limitation in this study. No direct associations were found among serum OPN levels, FEV_1_% predicted values, and MctPC20 levels. Longitudinal follow-up studies are necessary to clarify the exact role of OPN in the process of age-dependent changes in lung function and in the development of LOA. Taken together, respiratory viral infection and aging may induce OPN release, which induces TGF-β1/Smad3 signaling pathways, consequently enhancing airway inflammation and fibrosis in patients with LOA. Therefore, the serum OPN level may be a potential biomarker for the diagnosis of patients with LOA.

## Supplementary information

Supplementary materials
