# Supplementary material for: Osteopontin contributes to late-onset asthma phenotypes in adult asthma patients
Source: Exp Mol Med. 2020 Feb 3;52(2):253–65. doi: 10.1038/s12276-020-0376-2 (PMC7062758; doi:10.1038/s12276-020-0376-2)

**Osteopontin Contributes to Late-Onset Asthma Phenotypes in Adult Asthmatics**

(**Running title:** Osteopontin in Late-Onset Asthma)

Hoang Kim Tu, Trinh, MD^1,2^; Thuy Van Thao, Nguyen, MD, MS^3^, Seo-Hee, Kim,^4^; Thi Bich Tra, Cao, MD^4^; Quoc Quang, Luu, MD^4^; Seung-Hyun, Kim, PhD ^5^, and Hae-Sim, Park, MD, Ph.D^1,4^

1: Department of Allergy and Clinical Immunology, Ajou University Medical Center, Suwon, South Korea.

2: Center for Molecular Biomedicine, University of Medicine and Pharmacy at Ho Chi Minh city, Vietnam

3: Department of Pediatrics, University of Medicine and Pharmacy at Ho Chi Minh City, Vietnam.

4: Department of Biomedical Science, Ajou University School of Medicine, Suwon, South Korea

5: Translational Research Laboratory for Inflammatory Disease, Clinical Trial Center, Ajou University Medical Center, Suwon, South Korea

**Corresponding author**

Professor Hae-Sim, Park, MD, Ph.D.

Department of Allergy and Clinical Immunology, Ajou University School of Medicine, Yeongtong-gu, Suwon 443-380, South Korea

Tel: +82 31 219 5150, Fax: +82 31 219 5154

Email: [hspark@ajou.ac.kr](mailto:hspark@ajou.ac.kr)

**SUPPLEMENTARY TABLES**

**Table S1. Sequence of the primers used in this study**

| **No** | **Primer** | **Sequence** |
| --- | --- | --- |
| 1 | *Actin* | Forward: 5’-AGTGTGACGTTGACATCCGT-3’  Reverse: 5’-TGCTAGGAGCCAGAGCAGTA-3’ |
| 2 | *Areg* | Forward: 5’-CAGTGCACCTTTGGAAACGA-3’  Reverse: 5’-ATGTCATTTCCGGTGTGGCT-3’ |
| 3 | *Ch3l1* | Forward: 5’-AGGTTATCACCCCCATGACC-3’  Reverse: 5’-AGAAGCTTCCAACGCCTTCC-3’ |
| 4 | *Egfr* | Forward: 5’-GCCATCTGGGCCAAAGATAC-3’  Reverse: 5’-GGTGTGAGAGGTTCCACGAG-3 |
| 5 | *Il33* | Forward: 5’-TCACTGCAGGAAAGTACAGCAT-3’  Reverse: 5’-TGGGATCTTCTTATTTTGCAAGGC-3’ |
| 6 | *Spp1* | Forward: 5’-CCTGGCTGAATTCTGAGGGAC-3’  Reverse: 5’-ATCAGTCACTTTCACCGGGAG-3’ |
| 7 | *Tgfb1* | Forward: 5’-ACTGGAGTTGTACGGCAGTG-3’  Reverse: 5’-GGCTGATCCCGTTGATTTCC-3’ |

**SUPPLEMENTARY FIGURE LEGENDS**

**Fig. S1. Cytokines released from epithelial cells stimulated with Poly(I:C)** (a), IL-8 (b), TGF-β1, and (c) CH3L1 released from HAEC treated with Poly (I:C). In some experiments, cells were primed with Dex, Mon, and anti-IL-5 antibody. *P* values were analyzed by Mann-Whitney U test. **, ***, *P*<0.01, <0.001 compared between groups. Data was shown as log-transformed, mean ± SD.

CH3L1, chitinase 3-like 1; Dex, dexamethasone; Mon, montelukast; IL, interleukin; Poly(I:C), polyinosinic: polycytidylic; TGF-β1, transforming growth factor β1.

**Fig S2. Histological analysis of lung tissues** Lung tissues were harvested, fixed, and then embedded in paraffin for histology. A 10 μm thickness slide was prepared for each sample. Tissues were stained with hematoxylin-eosin (HE) for inflammatory cell count; periodic-acid Schiff (PAS) for mucus producing cells and Masson’s Trichrome (MT) for visualization of fibrosis. Representative images were shown. (a) Age-associated changes; (b) effects of OPN and (c) anti-asthmatic treatments on histological characteristics.

CH3L1, chitinase 3-like 1; Dex, dexamethasone; Mon, montelukast; IL, interleukin; OPN, osteopontin; Poly(I:C), polyinosinic: polycytidylic; TGF-β1, transforming growth factor β1.

**Fig S3. Analysis of histological data** (a) Inflammatory cell count, (b) % of mucus-positive area, (c) % of collagen area and (d) smooth muscle thickness were calculated. *P* values were analyzed by Mann-Whitney U test.

CH3L1, chitinase 3-like 1; Dex, dexamethasone; Mon, montelukast; IL, interleukin; OPN, osteopontin; Poly(I:C), polyinosinic: polycytidylic; TGF-β1, transforming growth factor β1.


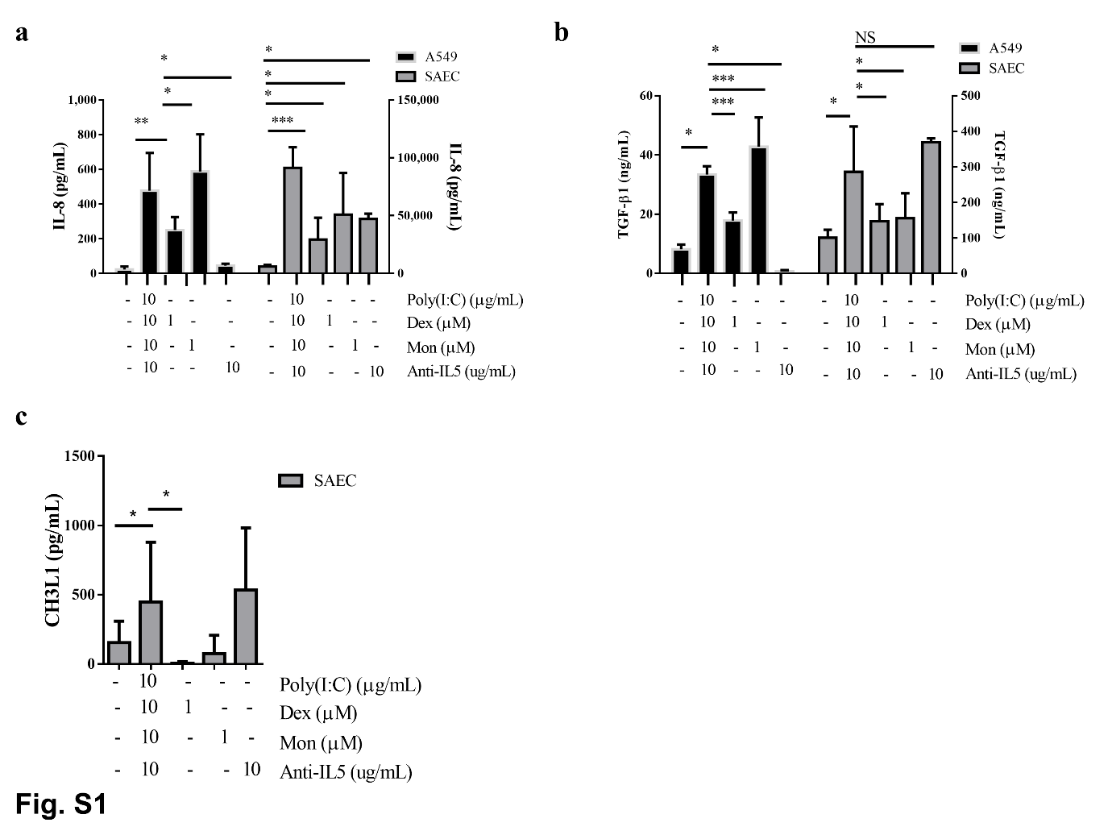
**SUPPLEMENTARY FIGURES**


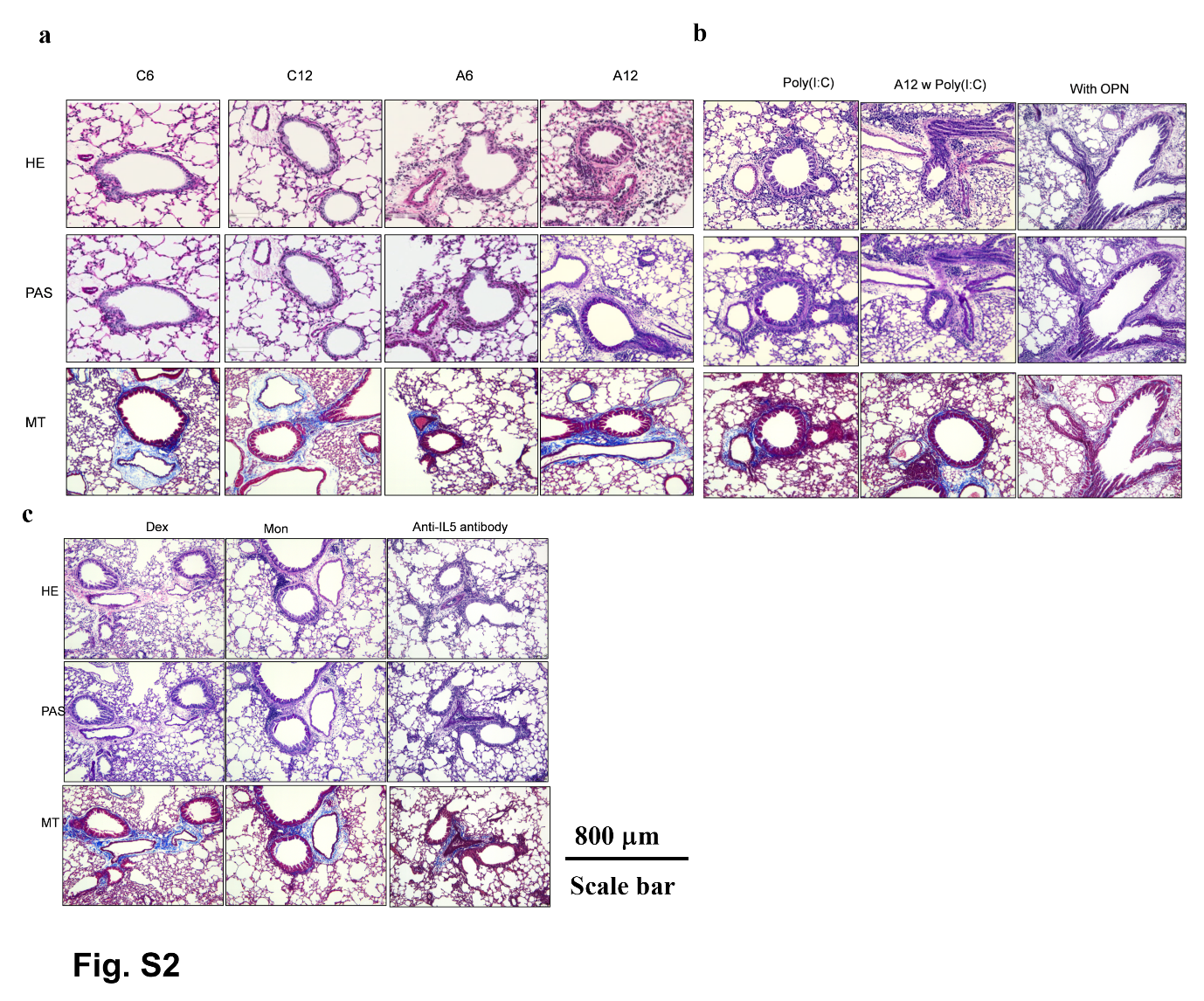

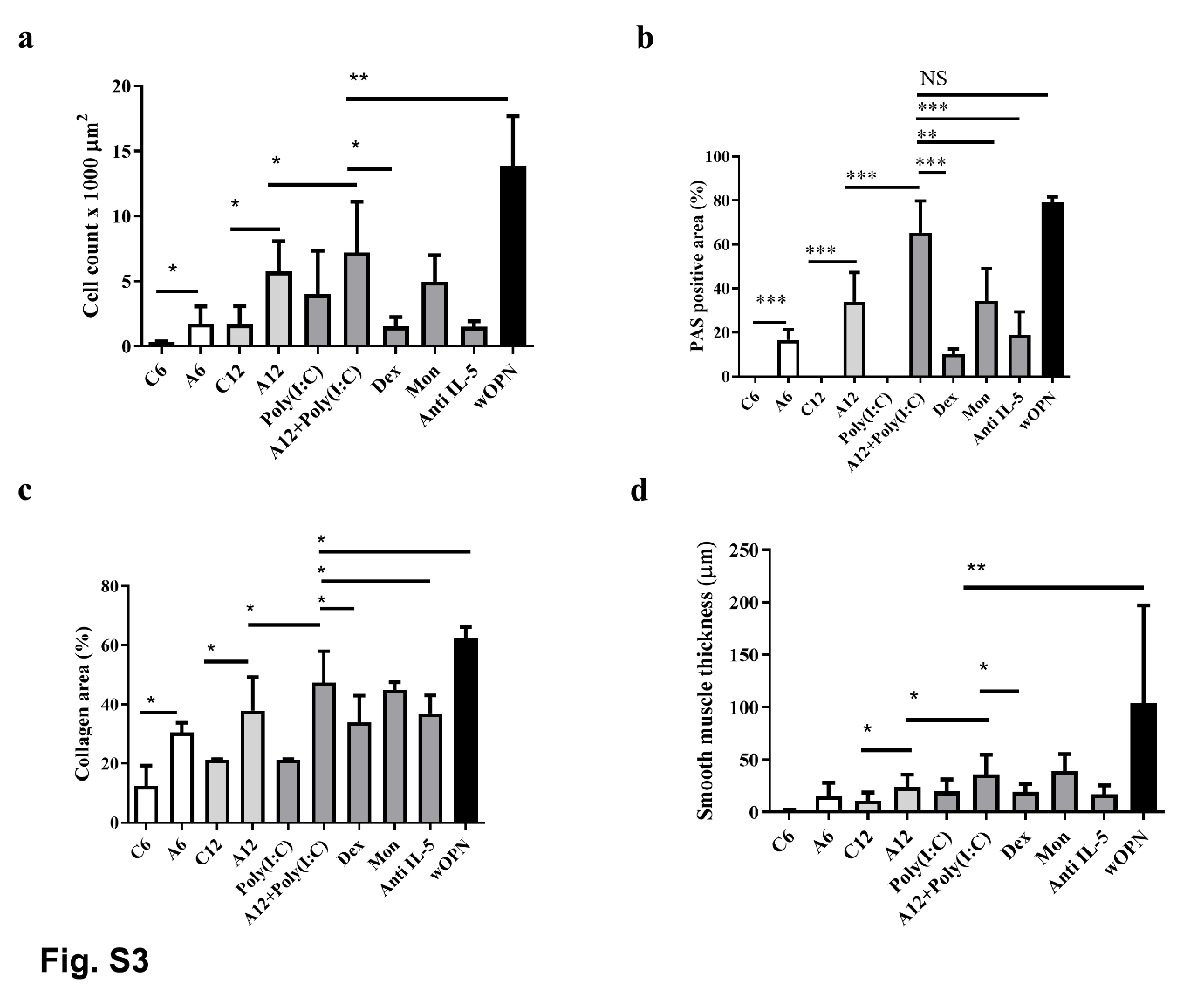

Supplement: Supplementary file 1 — Supplementary materials [file 12276_2020_376_MOESM1_ESM.docx]
